# Myocardial infarction and mortality following joint surgery in patients with rheumatoid arthritis: a retrospective cohort study

**DOI:** 10.1186/s13075-016-0958-5

**Published:** 2016-03-28

**Authors:** Joanne Tropea, Caroline A. Brand, Megan Bohensky, Sharon Van Doornum

**Affiliations:** Melbourne EpiCentre, The Royal Melbourne Hospital and University of Melbourne, Grattan Street, Parkville, VIC 3050 Australia; Department of Epidemiology and Preventive Medicine, Monash University, 99 Commercial Road, Melbourne, VIC 3004 Australia

**Keywords:** Rheumatoid arthritis, Joint surgery, Myocardial infarction, Mortality, Cardiovascular risk, Perioperative complications

## Abstract

**Background:**

Rheumatoid arthritis (RA) is associated with an increased risk of myocardial infarction (MI) and post-MI fatality compared with the general population. In a previous study examining post-MI treatment in RA compared with controls we noted that a higher proportion of the RA patients had experienced MI following a surgical procedure. The aim of this study was to compare the risk of MI and mortality at 6 weeks and 12 months following joint surgery in patients with RA compared with the general population.

**Methods:**

Individuals who had undergone joint surgery in Victoria, Australia between 1 July 2000 and 30 June 2007 were identified from routinely collected hospital administrative data. Logistic regression analysis was performed to examine odds of 6 week and 12 month MI and mortality in RA versus non-RA patients with adjustment for age, sex, comorbidities, socioeconomic status, patient type and admission type. Subgroup analysis of total hip and knee arthroplasty episodes was undertaken.

**Results:**

A total of 308,589 episodes of joint surgery occurred among 240,571 individuals, with 3654 (1.2 %) occurring among patients with RA. At 6 weeks post joint surgery the adjusted odds ratio (OR) for MI was 1.50 (95 % CI 0.96–2.33), all-cause death was 1.85 (95 % CI 1.09–3.13) and cardiovascular death was 1.90 (95 % CI 1.07–3.37). At 12 months post joint surgery the adjusted OR of MI was 1.70 (95 % CI 1.27–2.28), all-cause death was 2.18 (95 % CI 1.66–2.86) and cardiovascular death was 2.30 (95 % CI 1.65–3.22). On analysis of joint surgeries other than hip or knee arthroplasty, people with RA were at greater risk of MI within 6 weeks (adjusted OR 2.32; 95 % CI 1.24–4.34) and 12 months (adjusted OR 2.20; 95 % CI 1.47–3.30) compared to those without RA, but no difference in odds of short term mortality were found.

**Conclusions:**

Following an episode of joint surgery RA patients have a significantly increased risk of death at 6 weeks, and MI and death at 12 months, compared to the general population. The reasons for this remain to be elucidated but in the meantime RA patients should be considered at higher risk in the perioperative period.

**Electronic supplementary material:**

The online version of this article (doi:10.1186/s13075-016-0958-5) contains supplementary material, which is available to authorized users.

## Background

Rheumatoid arthritis (RA) is the most common form of inflammatory arthritis affecting up to 1.3 % of people worldwide and approximately 2 % of Australians [1, 2]. RA is associated with excess cardiovascular mortality and morbidity, which contributes significantly to the burden of this disease [3–5].

In an earlier study investigating mortality following a first acute cardiovascular event we found RA patients had increased 30-day case fatality after acute myocardial infarction (MI) compared to the general population [4]. Subsequent interrogation of hospital data from three Victorian tertiary referral centres showed that in patients who experienced MI, a preceding medical illness or surgical event was more substantially common among patients with RA than in those without RA (31 % vs 16 %, respectively) [6]. In other words, in almost a third of the RA patients who had MI, the MI had been preceded and possibly precipitated by a medical or a surgical *insult*. This led us to postulate that patients with RA might have an increased burden of silent cardiovascular disease, which is uncovered by a period of physiologic stress. To examine this further we wished to measure the risk of MI and other adverse cardiovascular outcomes in patients with RA compared to the general population following some form of physiologic insult. We chose joint surgery to represent our physiologic insult given that is a relatively standardized procedure that is not uncommonly performed in patients with RA.

Hence, the primary aim of this retrospective cohort study was to compare the risk of MI and death within 6 weeks and 12 months of any form of joint surgery in patients with RA versus patients without RA. The limited research into perioperative cardiovascular risk among patients with RA is primarily based on examination of cardiovascular complications following total knee joint replacement (TKJR) and total hip joint replacement (THJR), so we additionally performed subgroup analyses in these groups to facilitate direct comparison [7–10].

## Methods

### Data sources

In the Australian state of Victoria, data pertaining to all hospital admissions are collected within the Victorian Admitted Episodes Dataset (VAED), which is maintained by the Victorian Department of Health and Human Services. This dataset contains demographic and clinical information on each hospital admission, with the clinical information coded in the format of the International Statistical Classification of Diseases and Related Health Problems, Tenth Revision, Australian Modification (ICD-10-AM) [11]. Coding is based on review of the entire record of the hospital admission, carried out by experienced coders who obtain clinician input in the event of uncertainty. Up to 40 clinical codes and 40 procedural codes can be recorded for each hospital admission. The Victorian Department of Health maintains VAED data quality with an annual independent audit cycle, which samples nearly 1 % of public hospital discharges [12]. The Victorian Death Index is maintained by the Victorian Registry of Births, Deaths and Marriages and is a record of all certified deaths. Since 1 July 1996, statistical linkage techniques have been used to match records from the VAED to the Death Index. The linked data formed the basis of our study.

### Study population and definitions

All admissions with a procedure code indicating joint surgery (see Additional file 1) between 1 July 2000 and 30 June 2007 were included in the primary analysis. Subgroup analysis of THJR, TKJR episodes and all other joint surgeries was also undertaken. Data for at least 2 years prior to, and 12 months following, the joint surgery admission were available to identify patients with RA (with a 2-year retrospective period) and end points of interest as outlined below. As such, the total dataset spanned the period 1 July 1998 to 30 June 2008. Individuals with RA were identified by the presence of an ICD-10-AM code for RA (M05xx; M060x; M062x; M063x; M068x; M069x) during the joint surgery admission or during any hospital admission in the 2-year retrospective period.

### Outcomes

MI was defined by the ICD-10-AM diagnostic code I21xx, and occurrence of MI within 6 weeks and 12 months of joint surgery was generated from the index joint surgery admission and hospital admissions that occurred in the 12 months following the index admission. Death within 6 weeks and 12 months of joint surgery was generated from the death registry component of the dataset. Cause of death data were provided in text format, and a text string-searching algorithm was used to search cause of death data for cardiovascular deaths. For the purposes of this study, cardiovascular death included the following causes: MI, angina pectoris, ischemic heart disease, congestive heart failure, cardiomyopathy, arrhythmia, pulmonary edema, stroke, thromboembolism and cardiac arrest.

### Other covariates

Individual comorbidities from the ICD-10-AM version of the Charlson Comorbidity Index [13] and additional comorbidities of clinical significance (hypertension, smoking and dyslipidemia) were defined using ICD-10-AM diagnostic codes recorded at the time of joint surgery (Additional file 1). Socioeconomic status was estimated using the Index of Relative Socioeconomic Disadvantage (IRSD) of the Socio-Economic Indexes for Areas (SEIFA) based on place of residence (statistical local area), from the Australian Bureau of Statistics 2006 Census data [14]. The IRSD is derived from the Census variables related to disadvantage, such as low income, low educational attainment, unemployment, and dwellings without motor vehicles. A dichotomous variable was generated from the IRSD, reflecting statistical local areas in the lowest quartile in comparison to the areas in the top three quartiles.

The Accessibility/Remoteness Index of Australia (ARIA) from the Australian Department of Health and Aged Care was used to provide an indication of availability of goods and services, including health services, in an individual’s statistical local area [15]. The ARIA methodology uses Geographical Information System capabilities to produce a continuous variable with values between 0 and 12, where 0 indicates areas of highest accessibility and 12 indicates areas of highest remoteness. Those areas classified as highly accessible have no restrictions on their access to most goods and services. In Victoria, which has fewer remote regions compared to other parts of Australia, the ARIA score corresponds to three levels of accessibility: highly accessible (ARIA score 0–1.84), accessible (ARIA score >1.84–3.51) or moderately accessible (ARIA score >3.51–5.80). For this study, a dichotomous variable was generated from the ARIA comparing those living in highly accessible areas to those living in accessible or moderately accessible areas.

Patients who are admitted to a Victorian hospital are classified as either public, private, Department of Veterans Affairs (DVA) or compensable (work-related injury or transport accident) based on the funding source for the hospital admission. In Australia, all eligible residents have access to medical care through Medicare, the publicly funded universal health care scheme [16]. Medicare covers in-hospital inpatient and outpatient treatment at no cost to the patient, and subsidized community medical care, investigations and pharmaceuticals. Australians can chose to have Medicare cover only, or a combination of Medicare (public) and private health insurance. Medicare does not cover private patient hospital costs. A dichotomous variable was generated to compare public patients to all other patient types.

### Statistical methods

The statistical analysis consisted of initial descriptive and exploratory bivariate analysis, comparing demographic characteristics and clinical features of those with RA to those without. All categorical data were assessed using the Chi-square test, and the Mann–Whitney test was used to assess the difference in age between the two groups.

Multivariate logistic regression analyses were conducted to assess the impact of RA on MI and mortality outcomes after adjusting for relevant covariates. Four logistic regression models were fitted with RA status as the key exposure variable and outcomes, including 6-week and 12-month postoperative MI, all-cause mortality and cardiovascular mortality, respectively. Covariate adjustments included age, sex, comorbidities, socioeconomic status and type of joint surgery (hip and knee arthroplasty vs all other types of surgery), and variables with a *p* value <0.2 in the bivariate analysis were included [17]. Robust standard errors were used to allow for clustering by patient, as some patients had multiple episodes during the study period [18]. Statistical significance was set at *p* <0.05. All analyses were conducted using Stata/SE 12.1 software (StataCorp LP, TX, USA). This study was approved by the Melbourne Health Human Research Ethics Committee (HREC number 2010.104).

## Results

During the study period 240,571 individuals underwent a total of 308,589 joint surgery procedures, of which 3,654 (1.2 %) were performed on 2,219 people (0.92 %) with RA. Among the RA group, hip and knee joint arthroplasty were the most commonly performed surgical procedures (55.5 %), whereas in the non-RA group these types of surgery accounted for 26.6 % of all procedures. See Additional file 2 for further details of type of joint surgery episodes by RA status. The demographic details and clinical features of the RA and non-RA groups are shown in Table 1. Compared to those without RA, the patients with RA were older and more likely to be female, of lower socioeconomic status, less likely to be smokers and had a greater burden of comorbid disease, including hypertension, diabetes, vascular disease, pulmonary disease and renal disease. There were fewer elective and more public patient admissions among people with RA compared to those without RA, but there were no differences between the groups in accessibility to goods and services, or the numbers with an English-speaking background. See Additional file 3 for summary of patient level data.

**Table 1:** Summary of joint surgery admissions among patients with and without rheumatoid arthritis (RA)

	RA	Non-RA	*p* value
Joint surgery admissions, n (%)	3654 (1.2)	304,935 (98.8)	
Female, n (%)	2727 (74.6)	139,184 (45.6)	<0.0001
Age, median years (interquartile range)	64 (54–72)	54 (39–67)	<0.0001
Comorbidities during joint surgery admission, n (%)			
Smoker	386 (10.6)	40,958 (13.4)	<0.0001
Hypertension	308 (8.4)	16,269 (5.3)	<0.0001
dyslipidaemia	39 (1.1)	2612 (0.86)	0.170
Diabetes mellitus	218 (6.0)	12,682 (4.2)	<0.0001
Peripheral vascular disease	16 (0.44)	410 (0.13)	<0.0001
Pulmonary disease	74 (2.0)	1892 (0.62)	<0.0001
Peptic ulcer disease	12 (0.33)	269 (0.09)	<0.0001
Renal disease	56 (1.5)	1744 (0.57)	<0.0001
Liver disease	2 (0.05)	104 (0.03)	0.504
Cerebrovascular accident	21 (0.57)	787 (0.26)	<0.0001
Congestive heart failure	56 (1.5)	1673 (0.55)	<0.0001
Cancer or metastatic cancer	15 (0.14)	844 (0.28)	0.127
Paraplegia	16 (0.44)	466 (0.15)	<0.0001
Dementia	6 (0.16)	333 (0.11)	0.318
Human immunodeficiency disease	0	40 (0.01)	0.489
Lowest quartile of socioeconomic status^a^, n (%)	446 (12.2)	32,312 (10.6)	0.002
High accessibility to goods and services^b^, n (%)	3214 (88.0)	269,242 (88.3)	0.301
Public hospital patient, n (%)	1343 (36.8)	76,246 (25.0)	<0.0001
Elective admission, n (%)	3428 (93.8)	294,892 (96.7)	<0.0001
English-speaking country of birth, n (%)	3119 (85.4)	259,100 (85.0)	0.512
Hip and knee total, partial or revision arthroplasty, n (%)	2028 (55.5)	81,094 (26.6)	<0.0001

^a^Index of Relative Socioeconomic Disadvantage from the Australian Bureau of Statistics 2006 Census data; ^b^Accessibility Remoteness Index of Australia

Table 2 shows the number of events and odds ratios (OR) of MI and mortality following joint surgery in the RA and non-RA groups. Unadjusted models showed those with RA had greater odds of experiencing MI or death at both 6 weeks and 12 months compared to the non-RA group. After adjusting for age, sex, comorbidities and other potential confounders the OR for MI within 6 weeks of joint surgery in the RA patient group remained elevated but was no longer statistically significant (OR 1.50, 95 % CI 0.96–2.33) (Table 2). The adjusted OR for all other 6-week and 12-month outcomes remained statistically higher in the RA group as follows: all-cause death within 6 weeks (adjusted OR 1.85, 95 % CI 1.09–3.13) and 12 months (adjusted OR 2.18, 95 % CI 1.66–2.86), cardiovascular death within 6 weeks (adjusted OR 1.90, 95 % CI 1.07–3.37) and 12 months (adjusted OR 2.30, 95 % CI 1.65–3.22) and MI within 12 months (adjusted OR 1.70, 95 % CI 1.27–2.28).

**Table 2:** Myocardial infarction and mortality following joint surgery in patients with and without rheumatoid arthritis

Outcome	RA (*n* = 3654)	Non-RA (*n* = 304,935)	Unadjusted OR^a^ (95 % CI)	Adjusted OR^ab^ (95 % CI)
MI within 6 weeks	25 (0.68)	838 (0.27)	*2.50 (1.68–3.73)*	*1.50 (0.96–2.33)*
MI within 12 months	59 (1.61)	1968 (0.65)	*2.53 (1.95–3.28)*	*1.70 (1.27–2.28)*
All-cause death within 6 weeks	22 (0.60)	569 (0.19)	*3.24 (2.11–4.97)*	*1.85 (1.09–3.13)*
All-cause death within 12 months	93 (2.55)	2411 (0.79)	*3.28 (2.66–4.04)*	*2.18 (1.66–2.86)*
CV death within 6 weeks	15 (0.41)	372 (0.12)	*3.37 (2.01–5.66)*	*1.90 (1.07–3.37)*
CV death within 12 months	61 (1.67)	1406 (0.46)	*3.67 (2.83–4.75)*	*2.30 (1.65–3.22)*

Values for RA and Non-RA are the number (%) of episodes. ^a^Reference group patients without rheumatoid arthritis; ^b^adjusted for age, sex, comorbidities, admission type (emergency vs elective), patient type (public vs private), socioeconomic status and joint surgery type (hip, knee, ankle and shoulder joint arthroplasty/arthrodesis vs all other). *MI* myocardial infarction, *CV* cardiovascular, *RA* rheumatoid arthritis, *O*, odds ratio; *CI* confidence interval. Results in italics are statistically significant

The results from the subgroup analysis are given in Table 3. Among the THJR group, the adjusted OR for CV death within 6 weeks and 12 months, and all-cause death within 12 months were significantly higher for those with RA. Following TKJR surgery, the adjusted OR for MI and death at 6 weeks and 12 months were no different between the groups. Following all other types of joint surgery, the adjusted OR for MI at 6 weeks and all 12-month outcomes was significantly higher for those with RA. However, the adjusted OR for death within 6 weeks was no different between the RA and non-RA group.

**Table 3:** Adjusted odds ratios for myocardial infarction and mortality following THJR, TKJR and all other joint surgery in patients with and without rheumatoid arthritis

Outcome	THJR: 35,179 episodes	TKJR: 32,787 episodes	All other joint surgery: 240,633 episodes
	Adjusted OR^a^ (95 % CI)	Adjusted OR^a^ (95 % CI)	Adjusted OR^a^ (95 % CI)
MI within 6 weeks	1.31 (0.56–3.05)	0.84 (0.33–2.14)	2.32 (1.24–4.34)
MI within 12 months	1.56 (0.87–2.79)	1.22 (0.71–2.11)	2.20 (1.47–3.30)
All-cause death within 6 weeks	2.13 (0.93–4.90)	1.38 (0.27–7.04)	1.84 (0.90–3.76)
All-cause death within 12 months	1.79 (1.07–2.99)	1.57 (0.79–3.13)	2.72 (1.92–3.84)
CV death within 6 weeks	2.58 (1.02–6.52)	0.89 (0.12–6.68)	1.93 (0.85–4.36)
CV death within 12 months	1.99 (1.09–3.60)	1.86 (0.87–4.00)	2.70 (1.76–4.15)

^a^Reference group patients without rheumatoid arthritis. *THJR* total hip joint replacement, *TKJR* total knee joint replacement, *MI* myocardial infarction, *CV* cardiovascular, *RA* rheumatoid arthritis, *OR* odds ratio, *CI* confidence interval. Adjusted for age, sex, comorbidities, socioeconomic status, patient type and admission type

## Discussion

This large population-based study investigated the 6-week and 12-month risk of MI and mortality among RA patients undergoing any form of joint surgery, with subgroup analysis of total hip and knee arthroplasty. Patients with RA had adjusted odds of cardiovascular and all-cause mortality at 6 weeks after surgery that were nearly twice those of patients without RA. In our subgroup analysis, patients with RA had significantly higher odds of MI within 6 weeks and 12 months following joint surgery other than TKJR and THJR. An increased risk of early cardiovascular mortality was also demonstrated when analysis was limited to THJR surgery but not in patients with RA who underwent TKJR or other types of joint surgery. Outcomes at 12 months were almost universally worse for patients with RA, with cardiovascular and all-cause mortality significantly elevated in the cohort overall, and in the subgroup analysis among those who had undergone THJR surgery and joint surgery other than hip and knee arthroplasty. MI within 12 months of surgery was increased in the RA group overall, and was elevated but not statistically significantly so for the patients with RA who underwent total hip or knee joint arthroplasty.

Our findings of an increased risk of short-term death in the overall joint surgery cohort are consistent with a previous study. Soohoo et al. reviewed data from 138,399 patients undergoing primary THJR in California from 1995 to 2005, and as in our study, found that patients with RA had a significantly increased risk of short-term (90-day) mortality compared to patients with osteoarthritis (OR 1.88, 95 % CI 1.17–3.03) [19]. However, in most studies, as with the findings from our subgroup analysis of TKJR and THJR surgery, there was no difference in all-cause short-term mortality following hip and knee arthroplasty [7–9, 20–23]. A meta-analysis by Ravi et al. reported no evidence of excess 90-day mortality in patients with RA following THJR (OR 1.40, 95 % CI 0.82–2.39), or TKJR (OR 0.86, 95 % CI = 0.66–1.12) [21]. Subsequent to the meta-analysis, a number of studies have examined complications, including cardiovascular events and mortality, in patients with RA, following total joint arthroplasty. All studies reported no difference in the odds of short-term mortality [7, 8, 22, 23].

Very few studies have investigated long-term risk of mortality. We found a significant difference in the odds of 12-month mortality between the RA and non-RA groups in the total cohort or in the THJR subgroup analysis. Michaud et al. analyzed 7 years of data from US veterans to examine the complications and mortality in RA patients following total hip or knee arthroplasty [22]. Unlike our findings, there was no difference identified in mortality at 12 months (OR 1.30, 95 % CI 0.87–1.96) in the 839 patients with RA compared to those with osteoarthritis. However in their analysis of mortality up to 8 years post-surgery they reported an increased risk among those with RA compared to those with osteoarthritis (hazard ratio 1.22, 95 % CI 1.00–1.49).

Short-term risk of cardiovascular complications other than death has been assessed in a number of studies. In our total cohort of patients who underwent joint surgery and among those who underwent THJR and TKJR we found there was no difference in short-term risk of MI between the RA and non-RA groups. Similarly, in patients with RA compared to those with osteoarthritis, Michaud et al. did not observe any difference in the odds of cardiovascular events (including MI and cerebrovascular accident) within 30 days. In another study of 351,103 TKJR episodes between 2006 and 2010, of which 11,755 patients had RA, Stundner and colleagues also did not find any difference in the odds for combined perioperative adverse events (which included 30-day mortality, MI, infections and other complications) in patients with RA compared to those with osteoarthritis (adjusted OR 0.98, 95 % CI 0.92–1.05) [7]. In contrast, Stundner et al. examined US data from 157,775 THJR episodes between 2006 and 2010, of which 5,400 were in patients with RA, and found that patients with RA had higher overall odds of perioperative complications, including cardiovascular complications (adjusted OR 1.15, 95 % CI 1.05–1.25), compared to people with osteoarthritis [8].

Among those who underwent surgery other than THJR and TKJR, we found people with RA had an increased risk of MI within 6 weeks. This group of patients were mainly undergoing knee arthroscopy (69 %) and surgery on the shoulder (16 %) with the remainder undergoing hand/wrist, elbow and ankle surgery (8 %) and a small number undergoing revision arthroplasty of the hip or knee (6 %). This increased risk of MI within a group predominantly undergoing a presumably less significant hemodynamic insult is of interest and suggests the possibility that even relatively minor physiologic stress could precipitate a cardiovascular event in a predisposed patient with RA. Our data do not specifically address this issue, however.

The differences in findings from our study compared to other studies may be explained by a number of factors, including the differences in study time periods examined, the prevalence of RA, the variation in the postoperative time period examined (for example 6 weeks versus 90 days), combination of cardiovascular complications into one measure in a number of studies (whereas we looked at MI on its own) and differences in demographic characteristics of the study population. In particular, the Michaud study was a predominantly male population compared to our study, which involved predominantly female patients with RA.

Our study adds to the findings of the above mentioned studies by reporting on outcomes for patients with RA following joint surgery other than hip and knee arthroplasty. We found that joint surgery was not related to short-term MI but was significantly associated with short-term mortality and long-term MI and mortality. It is unknown whether this finding is related to the overall increased risk of CV events and mortality among patients with RA [24]. Further research should consider an investigation of CV outcomes and mortality in patients with RA undergoing surgery and those not receiving surgery, to determine if there is an additive risk associated with surgery.

This study utilized 10 years of hospital administrative data for all types of joint surgery within a single Australian state and included outcome information for 308,589 procedures, including 3,654 involving patients with RA. There are, however, several limitations that should be noted. The classification of patients with RA was based on coded diagnosis of RA in the hospital record, which was not collected specifically for research purposes. We utilized a 2 year retrospective period to increase the chances of accurately detecting RA cases, however, mild cases of RA may have been missed. The use of hospital administrative data to ascertain RA status may result in misclassification. We may have misclassified people with mild RA as false negatives (that is, no diagnosis of RA), and only captured patients with more severe or active RA who had been hospitalized. However, this is likely to lead to a bias toward the null hypothesis and is therefore a more conservative estimation of the true effect. We did not have access to medication data or outpatient administrative data to further validate the RA diagnosis. However, findings from an Australian study into the quality of ICD-10 coding of Victorian hospital discharge data reported the sensitivity and positive predictive value (PPV) for identifying RA as 91 % (95 % CI 74–100 %) and 77 % (95 % CI 54–100 %), respectively [25]. In addition, a recent Canadian validation study reported the optimal algorithm for identifying patients with RA from health administrative data was one hospitalization code ever or three physician diagnosis codes from claims with one or more RA code by a specialist in a 2-year period [26]. The comparability of Canadian and Australian coded administrative hospital data for identifying RA is unknown. Our classification of comorbidities was also dependent on coded data, which may have inaccuracies but is unlikely to be differentially distributed between RA and non-RA cases, and therefore unlikely to introduce systematic bias into the results [27].

We were interested in assessing all-cause mortality as well as cardiovascular mortality. The cause of death data were available in text form only and did not allow us to use a more standardized approach (for example, using ICD-10 codes) to define cardiovascular death, or differentiate between an underlying and associated cause of death. We accept this is a limitation of the study as it may result in misclassification of this outcome. However, this is unlikely to be unequally distributed between the RA and non-RA group and again unlikely to introduce systematic bias into the results.

Information on RA severity, activity, treatment, preventive care and use of cardiovascular medicines was not available, and we therefore unable to examine the influence of these factors on postoperative mortality. Our dataset does not capture non-fatal events that occur outside the Victorian hospital setting, such as those that occur in the community and do not result in hospitalization or hospitalization in other Australian states. Consequently we may have underestimated the true burden of postoperative MI in our cohort. Finally, with the growing use of biological disease-modifying anti-rheumatic drugs, the incidence of joint surgery has been decreasing and long-term prognosis has been improving for people with RA [28, 29]. As such, the odds of mortality and morbidity presented in this study may differ from current measures of risk, as the profile of people with RA receiving joint surgery is evolving over time.

## Conclusion

In conclusion, at 6 weeks following joint surgery, patients with RA had almost double the odds of all-cause and CV mortality compared to those without RA. At 12 months post joint surgery, the RA group had significantly higher odds of MI, all-cause death and CV death compared to those without RA. Understanding the risks of postoperative MI and mortality can help patients with RA make informed decisions about undergoing joint surgery procedures. Further studies to determine the contribution of subclinical cardiovascular disease, RA disease severity, functional status and RA treatments to this increased mortality are warranted.

## Additional files

Additional file 1: International Statistical Classification of Diseases and Related Health Problems, Tenth Revision, Australian Modification (*ICD-10-AM*) codes used to identify joint surgery admissions and comorbidities. (DOCX 18 kb) Additional file 2: Breakdown of type of joint surgery episodes by rheumatoid arthritis status. (DOCX 16 kb) Additional file 3: Summary of patient level comparing those with rheumatoid arthritis to those without rheumatoid arthritis. (DOCX 14 kb)

## Abbreviations

ARIA: Accessibility/Remoteness Index of Australia
CI: confidence interval
CV: cardiovascular
DVA: Department of Veterans Affairs
ICD-10-AM: International Statistical Classification of Diseases and Related Health Problems, Tenth Revision, Australian Modification
IRSD: Index of Relative Socioeconomic Disadvantage
MI: myocardial infarction
OR: odds ratio
RA: rheumatoid arthritis
SEIFA: Socio-Economic Indexes for Areas
THJR: total hip joint replacement
TKJR: total knee joint replacement
VAED: Victorian Admitted Episodes Dataset
