# Supplementary material for: Myocardial infarction and mortality following joint surgery in patients with rheumatoid arthritis: a retrospective cohort study
Source: Arthritis Res Ther. 2016 Mar 28;18:69. doi: 10.1186/s13075-016-0958-5 (PMC4809028; doi:10.1186/s13075-016-0958-5)
Supplement: Additional file 1: — International Statistical Classification of Diseases and Related Health Problems, Tenth Revision, Australian Modification (ICD-10-AM) codes used to identify joint surgery admissions and comorbidities. (DOCX 18 kb) [file 13075_2016_958_MOESM1_ESM.docx]

**Additional file 1: ICD-10-AM codes used to identify joint surgery admissions and comorbidities**

| **ICD-10-AM Procedure code** | **Description** |
| --- | --- |
| Shoulder  48945-00  48948-01  48912-00  90600-00  48945-01  48948-00  48954-00  48951-00  48942-00  48957-00  48948-02  48915-00  48918-00  48960-00  48921-00  48924-00  Humerus or Elbow  49118-00  49100-00  49121-00  49121-01  49121-04  49118-01  49109-00  49121-02  49121-03  49112-00  49106-00  49115-00  90537-00  Wrist  49218-00  49221-00  49221-01  49212-00  49221-02  49218-01  49224-00  49224-01  49224-02  49227-00  49200-00  46324-00  49206-00  49209-00  90543-00 | Arthroscopy of shoulder  Arthroscopic removal of loose body of shoulder  Arthrotomy of shoulder  Arthroscopic release of adhesions or contracture of shoulder  Arthroscopic biopsy of shoulder  Arthroscopic debridement of shoulder  Arthroscopic synovectomy of shoulder  Arthroscopic decompression of subacromial space  Arthrodesis of shoulder with removal of prosthesis  Arthroscopic stabilisation of shoulder  Arthroscopic chondroplasty of shoulder  Hemiarthroplasty of shoulder  Total arthroplasty of shoulder  Arthroscopic reconstruction of shoulder  Revision of total arthroplasty of shoulder  Revision of total arthroplasty of shoulder with bone graft to scapula or humerus  Arthroscopy of elbow  Arthrotomy of elbow  Arthroscopic drilling of defect of elbow  Arthroscopic removal of loose body of elbow  Arthroscopic release of elbow contracture  Arthroscopic biopsy of elbow  Arthroscopic synovectomy of elbow  Arthroscopic chondroplasty of elbow  Arthroscopic osteoplasty of elbow  Silastic replacement of radial head of elbow  Arthrodesis of elbow  Total arthroplasty of elbow  Revision arthroplasty of elbow  Arthroscopy of wrist  Arthroscopic drilling of defect of wrist  Arthroscopic removal of loose body of wrist  Arthrotomy of wrist  Arthroscopic release of adhesions of wrist  Arthroscopic biopsy of wrist  Arthroscopic debridement of wrist  Arthroscopic synovectomy of wrist  Arthroscopic osteoplasty of wrist  Arthroscopic pinning of osteochondral fragment of wrist  Arthrodesis of radiocarpal joint  Arthroplasty of carpal bone  Excision arthroplasty of wrist  Total arthroplasty of wrist  Revision arthroplasty of wrist  Excision arthroplasty of wrist  Total arthroplasty of wrist  Revision arthroplasty of wrist |
| Hand  46309-00  46312-00  46315-00  46318-00  46321-00  46309-01  46312-01  46315-01  46318-01  46321-01  46306-00  46306-01  46307-00  46307-01 | Arthroplasty of interphalangeal joint of hand, 1 joint  Arthroplasty of interphalangeal joint of hand, 2 joints  Arthroplasty of interphalangeal joint of hand, 3 joints  Arthroplasty of interphalangeal joint of hand, 4 joints  Arthroplasty of interphalangeal joint of hand, >= 5 joints  Arthroplasty of metacarpophalangeal joint, 1 joint  Arthroplasty of metacarpophalangeal joint, 2 joints  Arthroplasty of metacarpophalangeal joint, 3 joints  Arthroplasty of metacarpophalangeal joint, 4 joints  Arthroplasty of metacarpophalangeal joint, >= 5 joints  Interposition arthroplasty of interphalangeal joint of hand  Interposition arthroplasty of metacarpophalangeal joint  Volar plate arthroplasty of interphalangeal joint of hand  Volar plate arthroplasty of metacarpophalangeal joint |
| Hip  49360-00  49366-00  49303-00  49363-00  47522-00  49312-00  49315-00  90607-00  90607-01  49318-00  49319-00  49346-00  49324-00  49327-00  49330-00  49333-00  49339-00  49342-00  49345-00  Knee  49557-00  49500-01  49560-00  49560-02  49557-01  49558-00  49560-01  49566-00  49557-02  49560-03  49561-02  49562-02  49561-00  49562-00  49561-01  49562-01  49517-00  49518-00  49519-00  49534-01  49521-00  49521-01  49521-02  49521-03  49524-00  49524-01  49563-00  49558-01  49559-00  49558-02  49539-00  49542-00  49530-00  49530-01  49533-00  49554-00  49527-00  Ankle  49700-00  49706-00  49703-02  49700-01  49703-01  49703-04  49703-05  49703-03  49715-00  Other  50100-00  50103-00  50100-01  90608-01  50127-00  50102-00 | Arthroscopy of hip  Arthroscopic removal of loose body from hip  Arthrotomy of hip  Arthroscopic biopsy of hip  Hemiarthroplasty of femur  Excision arthroplasty of hip  Partial arthroplasty of hip  Resurfacing of hip, unilateral  Resurfacing of hip, bilateral  Total arthroplasty of hip, unilateral  Total arthroplasty of hip, bilateral  Revision of partial arthroplasty of hip  Revision of total arthroplasty of hip  Revision of total arthroplasty of hip with bone graft to acetabulum  Revision of total arthroplasty of hip with bone graft to femur  Revision of total arthroplasty of hip with bone graft to acetabulum and femur  Revision of total arthroplasty of hip with anatomic specific allograft to acetabulum  Revision of total arthroplasty of hip with anatomic specific allograft to femur  Revision of total arthroplasty of hip with anatomic specific allograft to acetabulum and femur  Arthroscopy of knee  Arthrotomy of knee  Arthroscopic removal of loose body of knee  Arthroscopic lateral release of knee  Arthroscopic biopsy of knee  Arthroscopic debridement of knee  Arthroscopic trimming of ligament of knee  Arthroscopic synovectomy of knee  Arthroscopic excision of meniscal margin or plica of knee  Arthroscopic meniscectomy of knee  Arthroscopic removal of loose body of knee with debridement, osteoplasty or chondroplasty  Arthroscopic removal of loose body of knee with chondroplasty and multiple drilling or implant  Arthroscopic lateral release of knee with debridement, osteoplasty or chondroplasty  Arthroscopic lateral release of knee with chondroplasty and multiple drilling or implant  Arthroscopic meniscectomy of knee with debridement, osteoplasty or chondroplasty  Arthroscopic meniscectomy of knee with chondroplasty and multiple drilling or implant  Hemiarthroplasty of knee  Total arthroplasty of knee, unilateral  Total arthroplasty of knee, bilateral  Total replacement arthroplasty of patellofemoral joint of knee  Total arthroplasty of knee with bone graft to femur, unilateral  Total arthroplasty of knee with bone graft to femur, bilateral  Total arthroplasty of knee with bone graft to tibia, unilateral  Total arthroplasty of knee with bone graft to tibia, bilateral  Total arthroplasty of knee with bone graft to femur and tibia, unilateral  Total arthroplasty of knee with bone graft to femur and tibia, bilateral  Arthroscopic repair of meniscus of knee  Arthroscopic chondroplasty of knee  Arthroscopic chondroplasty of knee with multiple drilling or implant  Arthroscopic osteoplasty of knee  Arthroscopic reconstruction of knee  Arthroscopic reconstruction of cruciate ligament of knee with repair of meniscus  Revision of total arthroplasty of knee with bone graft to femur  Revision of total arthroplasty of knee with bone graft to tibia  Revision of total arthroplasty of knee with bone graft to femur and tibia  Revision of total arthroplasty of knee with anatomic specific allograft  Revision of total arthroplasty of knee  Arthroscopy of ankle  Arthrotomy of ankle  Arthroscopic removal of loose body of ankle  Arthroscopic biopsy of ankle  Arthroscopic trimming of osteophyte of ankle  Arthroscopic synovectomy of ankle  Arthroscopic chondroplasty of ankle  Arthroscopic repair of osteochondral fracture of ankle  Total arthroplasty of ankle  Arthroscopy of joint, not elsewhere classified  Arthrotomy of joint, not elsewhere classified  Arthroscopic biopsy of joint, not elsewhere classified  Arthroscopic procurement of cartilage  Arthroplasty of joint, not elsewhere classified  Arthroscopic procedure of joint, not elsewhere classified |
| **ICD-10-AM Diagnosis codes** | **Description** |
| Z720; F171-F174  I10x  E780; E785 | *Smoking*  *Hypertension*  *Dyslipidaemia* |
