# Supplementary material for: Myocardial infarction and mortality following joint surgery in patients with rheumatoid arthritis: a retrospective cohort study
Source: Arthritis Res Ther. 2016 Mar 28;18:69. doi: 10.1186/s13075-016-0958-5 (PMC4809028; doi:10.1186/s13075-016-0958-5)
Supplement: Additional file 2: — Breakdown of type of joint surgery episodes by rheumatoid arthritis status. (DOCX 16 kb) [file 13075_2016_958_MOESM2_ESM.docx]

Additional file 2: Breakdown of type of joint surgery episodes by rheumatoid arthritis (RA) status*

| ***Type of joint surgery:*** | **RA, n (%)** | **Non-RA, n (%)** | **p value** |
| --- | --- | --- | --- |
| *Hip arthroplasty (total, partial and revision)* | 842 (23.0) | 41,341 (13.6) | <0.0001 |
| *Knee arthroplasty (total, partial and revision)* | 1190 (32.5) | 39,769 (13.0) | <0.0001 |
| *Other hip surgery without arthroplasty* | 2 (0.07) | 97 (0.04) | 0.348 |
| *Other knee surgery without arthroplasty* | 574 (15.7) | 165,402 (54.2) | <0.0001 |
| *Ankle surgery alone* | 81 (2.2) | 7896 (2.6) | 0.098 |
| *Ankle and knee surgery* | 0 | 215 (0.13) | 0.385 |
| *Shoulder and elbow arthroscopy, arthrodesis or arthroplasty* | 417 (11.4) | 41,607 (13.6) | <0.0001 |
| *Wrist and hand arthroplasty* | 457 (12.5) | 6947 (2.3) | <0.0001 |
| *Other* | 107 (2.9) | 2761 (0.9) | <0.0001 |

*Includes cases in which multiple types of joint surgery were undertaken during the one episode
