# Supplementary material for: Myocardial infarction and mortality following joint surgery in patients with rheumatoid arthritis: a retrospective cohort study
Source: Arthritis Res Ther. 2016 Mar 28;18:69. doi: 10.1186/s13075-016-0958-5 (PMC4809028; doi:10.1186/s13075-016-0958-5)
Supplement: Additional file 3: — Summary of patient level comparing those with rheumatoid arthritis to those without rheumatoid arthritis. (DOCX 14 kb) [file 13075_2016_958_MOESM3_ESM.docx]

Additional file 3: Summary of Patient level data comparing those with rheumatoid arthritis (RA) to those without rheumatoid arthritis (Non-RA)

|  | RA | Non-RA |
| --- | --- | --- |
| Number of patients | 2219 | 238,352 |
| Sex*, n (%):  Female  Male | 1643 (74.0)  576 (26.0) | 107,517 (45.1)  130,835 (54.9) |
| Age in years*, median (IQR) | 64 (54-73) | 53 (37-66) |
| English speaking country of birth*, n (%) | 1885 (85.0) | 201,378 (84.5) |
| Number of joint surgery admissions over study period, n (%)  1  2  3  ≥4 | 1435 (64.7)  520 (23.4)  157 (7.1)  107 (4.8) | 188,348 (79.0)  38,204 (16.0)  8376 (3.5)  3424 (1.4) |

*Summary of information from individuals’ first (index) joint surgery admission.
